# Core Synergies Measured with Ultrasound in Subjects with Chronic Non-Specific Low Back Pain and Healthy Subjects: A Systematic Review

**DOI:** 10.3390/s22228684

**Published:** 2022-11-10

**Authors:** Maria Cervera-Cano, Luis López-González, David Valcárcel-Linares, Samuel Fernández-Carnero, Alexander Achalandabaso-Ochoa, Verónica Andrés-Sanz, Daniel Pecos-Martín

**Affiliations:** 1 Health Technology Assessment Unit (UETS), 28046 Madrid, Spain; 2 Universidad de Alcalá, Facultad de Medicina y Ciencias de la Salud, Departamento de Enfermería y Fisioterapia, Grupo de Investigación en Fisioterapia y Dolor, 28801 Alcalá de Henares, Spain; 3 Department of Health Sciences, University of Jaén, 23071 Jaén, Spain; 4 Physiotherapy Support Unit, Barrio del Pilar Health Centre, 28029 Madrid, Spain

**Keywords:** nonspecific low back pain, ultrasound, abdominal muscles, synergism

## Abstract

Low back pain represents the leading cause of disability since 1990. In 90% of cases, it is classified as non-specific low back pain, being chronic in 10% of subjects. Ultrasound has proven to be an effective measurement tool to observe changes in the activity and morphology of the abdominal muscles. This article reviews which core synergies are studied with ultrasound in healthy subjects and with chronic non-specific low back pain. A systematic review was conducted on studies analyzing synergies between two or more core muscles. Publications from 2005 until July 2021 were identified by performing structured searched in Pubmed/MEDLINE, PEDro and WOS. Fifteen studies were eligible for the final systematic review. A total of 56% of the studies established synergies between the core muscles and 44% between the homo and contralateral sides of the core muscles. The most studied core synergies were transversus abdominis, internal oblique and external oblique followed by the rectus abdominis and the lumbar multifidus. No studies establishing synergies with diaphragm and pelvic floor were found. Eight studies were conducted in healthy subjects, five studies in subjects with chronic non-specific low back pain compared to healthy subjects and two studies in subjects with chronic non-specific low back pain.

## 1. Introduction

Low back pain has been the leading cause of disability since 1990 [1]. In 85–90% of cases, the exact cause of the pain cannot be determined with certainty and patients are classified as non-specific low back pain [1]. The estimated prevalence of non-specific low back pain (NSLBP) is 18%, which has a major socio-economic impact worldwide [2,3]. In 10% of people suffering from NSLBP, it becomes chronic [1], as it lasts longer than three months or is experienced for at least three full weeks of pain in the year [4]. The type of work, obesity and unfavorable habits in daily life have been considered risk factors that increase the likelihood of non-specific chronic low back pain [2]. It has been proposed that maladaptive social and psychological factors [depression, anxiety, catastrophizing and low self-efficacy) may play an important role in the persistence of pain [3,5]. However, the role of musculoskeletal factors remains unclear.

Although there is no consensus on an exact definition of the core, this term could be defined as the abdomino-pelvic functional unit that involves not only vertebral segments with their corresponding passive structures that support it or the neural system, but also the four fundamental pillars that compose it: lumbar multifidus (LM), lateral abdominal wall (LAW) formed by of the transversus abdominis (TrA), internal oblique (IO) and external oblique (EO), diaphragm (DF) and pelvic floor (PF) [6]. However, although the term core refers to several muscle groups, it should be considered a functional rather than an anatomical term [7,8]. This functional unit would act together, jointly, to provide the stability necessary to perform a task [9]. The musculature of the LAW and of the PF [10] maintains a close relationship with the lumbar and pubic regions. This musculature, together with the diaphragm [11], has a determining role in mechanical coordination [12] to ensure the stability of the tidal volume and the abdomino-lumbo-pelvic segment. For the action of this musculature of the cavity to be executed in a physiological and adequate manner, it is essential that there is harmony in the totality of the curves of the spine. In addition, the lumbar multifidus muscles are important stabilizers of the spine [7,8]. All these muscles form a cylinder that works in synergy, producing a crossroads of lumbo-pelvic forces that provide trunk stability, better trunk control, efficient movement, good balance, and coordination, as well as better postural firmness and alignment (motor control) [7,8].

Different tools have been used to assess muscle behavior and/or core morphology with electromyography (EMG) or magnetic resonance imaging (MRI) being the ‘Gold standard’. The use of these diagnostic tools has tried to reduce uncertainty about the presence or absence of NSLBP, to support a particular therapeutic management, as an aid in prognosis, or to monitor the clinical course of this disease. The use of rehabilitative ultrasound (RUSI) has proven to be more easily able to achieve these goals in a noninvasive, cost-effective way, and capable of distinguishing the different muscle groups separately and simultaneously [13]. The use of RUSI for the measurement of trunk musculature has shown good reliability with respect to MRI and EMG for the assessment of muscle activation and morphological changes [13]. The use of the motion mode of ultrasound has been shown to be a useful tool for assessing muscle activation in people with chronic NSLBP [6].

Through the use of the diagnostic tools mentioned above, several authors have drawn conclusions about musculoskeletal alterations, muscle activity and/or changes in core trophism in chronic NSLBP patients compared to asymptomatic participants [14]. It has been shown that a delay of activation of TrA [15], an atrophy of RA with an increased inter-rectus distance [16], LM atrophy accompanied by delayed activation or weaker contraction [17,18], excessive activation of the RA, IO and EO (higher percentage of muscle change during a dynamic task) [19], reduced diaphragmatic excursion with a higher position and increased fatigue which is compensated by an increase in lung volume to provide an adequate increase in intra-abdominal pressure [20]. In cases of women with chronic NSLBP, the absence of a local PF muscle activation strategy has been observed [21]. These changes appear to be important factors in the assessment and management of patients with low back pain [13].

In relation to the definition of the core and motor control, not only the morphological situation of the musculature and its activation in isolation seems to be important, but also the coordination between the muscle groups that compose this functional unit of the core [9]. This coordination or muscular activation relationship is defined as muscular synergies. An alteration of motor control and muscle synergies, being the basis for the correct functioning of the abdomino-pelvic area, could be a source of recurrent muscular nociceptive information.

Therefore, studies that analyze the global muscular behavior of the core and its morphological characteristics in a situation of pain in comparison with healthy subjects would provide a lot of information about the possible differences between both groups of subjects.

The aim of the present systematic review was to analyze the muscular synergies of the core in healthy subjects with chronic NSLBP measured by ultrasound that have been described in the scientific literature. Since the core is a functional unit, knowledge about its behavior is necessary. It is hypothesized that an alteration in this behavior as a functional unit may be related to the appearance or perpetuation of NSLBP. This review aims to draw conclusions about the global behavior of this musculature in healthy subjects and to compare it with subjects with NSLBP.

## 2. Materials and Methods

### 2.1. Literature Search

The systematic review was conducted according to the reporting guidance provided in the Preferred Reporting Items for Protocols for Systematic Reviews and Meta-Analyses (PRISMA-P 2020) [22]. The protocol of this systematic review is registered in OSF Registries (https://osf.io/hekfp accessed on 20 September 2022). DOI 10.17605/OSF.IO/VAWR3.

### 2.2. Eligibility Criteria

Observational studies, quasi-experimental studies, randomized or non-randomized clinical trials (RCTs) and systematic reviews or meta-analyses were included in the review. Additionally, studies that had been conducted in a hospital, clinical or academic setting, in any country, involving adult subjects ≥18 years, general population with chronic NSLBP, without pain or both. Studies of the reliability of ultrasound compared to other diagnostic tools (EMG, MRI, etc.), narrative reviews, opinion articles, studies that did not use ultrasound as the main measurement tool and/or that contained subjects with any other lumbar pathology such as fractures, infection, tumors or disc pathology were excluded.

### 2.3. Sources of Information

It was performed a comprehensive search for published studies indexed in PubMed/MEDLINE, PEDro and WOS from 2005 to July 2021. We also performed a manual search for additional relevant studies using references of the included articles.

### 2.4. Search Strategy

The search items include medical subject headings (MeSH) and the keywords: (“nonspecific low back pain”, “low back pain” and “lumbopelvic pain”), (“ultrasound”, “ultrasonography”), (“abdominal wall”, “abdominal muscles”, “multifidus”, “diaphragm,” “transversus abdominis” and “pelvic floor.”) and (“synergism”, “cocontraction”, “muscle contraction“ and “thickness”. These terms were combined using Boolean operators (“AND” or “OR”), and results from all the possible combinations were downloaded into an Zotero V.6.0.9 library (Corporation for Digital Scholarship, Vienna, VA, USA). Truncation and wildcard strategies were applied. The search was limited by the English language, but not to the study design or country of origin. Supplementary Section S1 describes the full search term used in each database searched.

### 2.5. Data Selection and Extraction Process

Zotero V.6.0.9 library (Corporation for Digital Scholarship) was used for an efficient management of the bibliography. Once the search was conducted and duplicates removed, two reviewers (M.C.C and L.L.G) independently examined the title and abstract for all potentially relevant citations. Duplicates and studies not relevant to title and abstract were excluded, and a full reading of the remaining articles was performed. Any disagreement was resolved by consensus or by a third reviewer (F.R.S.). This process was repeated at the results extraction stage.

The PRISMA-P 2020 flowchart [22] was used for the extraction process, representing the number of studies that were included or excluded and their causes. A data extraction form was developed using Microsoft® Excel 365 V.16.62 (22061100), Microsoft Corporation (Redmond, WA, USA) which ensures consistency of data extraction between review authors and contains data concerning the general details of the studies, such as their individual characteristics, participants, intervention, and study outcomes.

### 2.6. Outcomes

The primary outcome of our study included the muscle relationships or synergies of two or more muscles that compose the core represented as: muscle thickness patterns (cm, mm, ms or >/</=), onset (ms), muscle activation (% change in thickness (cm)) and symmetry (between the homolateral side with respect to the contralateral side) in percentage.

### 2.7. Risk of Bias Assessment

To assess the risk of bias of the studies included in the review, the Joanna Bridge Institute for Systematic Reviews (JBI) scale [23] was used for observational studies if they had a control group (see Supplementary Section S5: Table S4) and the Canadian Institute for Health Economics (IHE) scale [24] if they did not have a control group (see Supplementary Section S5: Table S5). The Cochrane Risk of Bias scale (Rob-2) was used for RCTs [25] (see Supplementary Section S4: Figure S1). Two authors (M.C.C and L.L.G) independently assessed each study for bias. Discrepancies were resolved by consensus.

## 3. Results

### 3.1. Selection of Studies

**Figure 1:**
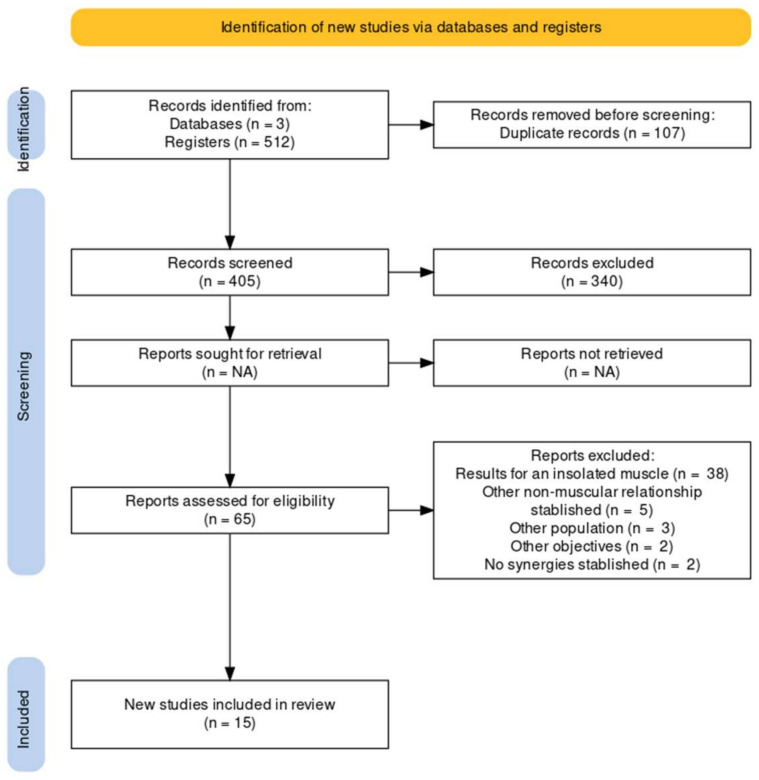
Study flowchart (PRISMA-P 2020).

After a comprehensive search for published studies indexed in Pubmed/Medline, WOS and PEDro databases, a total of 512 articles was retrieved by our search strategy. Subsequently, 497 studies that were duplicates, not relevant, had insufficient data, different population or had different objectives were excluded. A total of 15 articles met our inclusion criteria and were included in the systematic review. Two studies were RCTs [26,27] and 13 were observational case series (*n* = 8) [28,29,30,31,32,33,34,35] or observational case–control studies (*n* = 5) [36,37,38,39,40]. Figure 1 shows the selection process using the study flow chart (PRISMA-P 2020) [22]. All the excluded studies with their causes of exclusion can be found in Supplementary Section S3.

### 3.2. Characteristics of the Studies Included

To obtain the relevant information data was extracted such as type of population, intervention, comparator, objectives, measurement tools, type of synergy established, muscles studied and the outcomes.

The included studies (*n* = 15) [26,27,28,29,30,31,32,33,34,35,36,37,38,39,40] were divided according to their results. One study showed results for the two variables of interest in this review [20,31], so 15 studies were found but 16 synergies were described.

**Figure 2:**
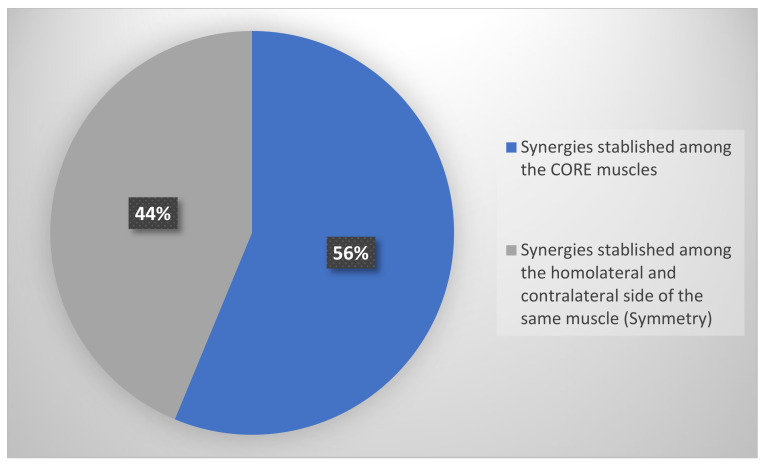
Types of synergies established with ultrasound.

Nine studies showed the synergies established among the core muscles [26,27,28,31,33,34,36,38,39] (56%) while the other seven studies showed results for synergies established between the homo and contralateral sides of the same muscle [30,31,32,33,35,37,40] described as symmetry (44%) (Figure 2).

The included studies (*n* = 15) [26,27,28,29,30,31,32,33,34,35,36,37,38,39,40] can be found below divided according to their results in Table 1 and Table 2. These tables are designed to show the main results of each study based on the core synergies established in each study according of the objective of this review. Table 1 shows the main results of synergies established among the core muscles (*n* = 9) and Table 2 presents the results on the synergies established between the homolateral and contralateral side of the same muscle within the core region. The complete data extracted from the included studies can be found in Supplementary Section S2: Table S2.

### 3.3. Outcomes

#### 3.3.1. Outcomes: Synergies Established among the Core Muscles Measured with Ultrasound

In nine studies [26,27,28,31,33,34,36,38,39] that provided results on the synergies established between the core muscles measured by ultrasound, the TrA was the muscle that showed the most results. TrA was related in several studies to the other four muscle groups (IO, EO, RA and LM), either as muscle activation (% change in thickness) (*n* = 4), [26,28,38,39], muscle pattern (*n* = 3) [27,31,33], onset (*n* = 1) [34] or as tissue deformation rate (TDI) (*n* = 1) [36].

In five of the nine studies, results of synergies among the muscles that compose the lateral abdominal wall were shown (TrA, IO, EO) [27,34,36,38,39]. One of the nine studies established muscle activation relationships of each of the lateral abdominal wall muscles (TrA, IO and EO) with the LM [38], while another study relates only TrA to LM [26]. In two of the nine studies, RA was related to each of the muscles of the LAW [31,33], while another study relates RA to TrA activation [28].

**Figure 3:**
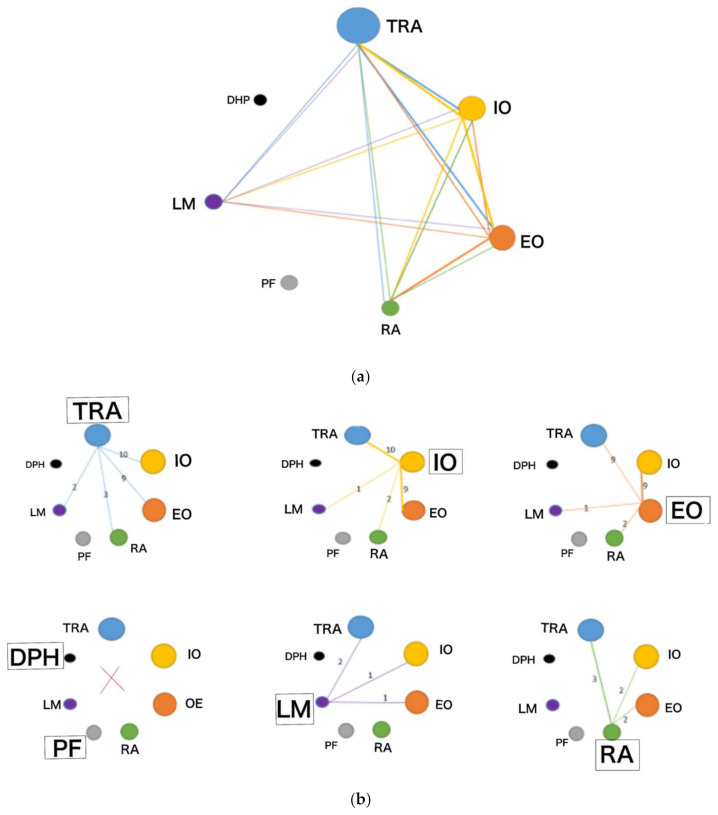
(**a**). Synergies established among the core muscles: Transversus abdominis: TrA; internal oblique: IO; external oblique: EO; rectus abdominis: RA; pelvic floor: PF; lumbar multifidus: LM; diaphragm: DPH. (**b**). Synergies established between the core muscles. Transversus abdominis: TrA; internal oblique: IO; external oblique: EO; rectus abdominis: RA; pelvic floor: PF; diaphragm: DPH; lumbar multifidus: LM.

No studies were identified that show results of muscle synergies measured with ultrasound for the pelvic floor musculature (PF) or diaphragm (DPH), nor were any studies identified that related muscle activation of the seven muscle groups that composes the functional unit of the core with ultrasound (RA, TrA, IO, EO, DF, PF and LM). These results are shown in Figure 3a,b.

#### 3.3.2. Synergies Established among the Homolateral and Contralateral Side of the Same Muscle with Ultrasound (Symmetry)

There were seven studies that assessed symmetry among the homolateral and contralateral sides of the same muscle. Three of the seven studies showed symmetry results for TrA, IO and EO [30,32,35]. Two of the seven studies showed symmetry results for the TrA, IO, EO and RA [31,33]. Additionally, two of the seven studies showed symmetry results for TrA and IO [37,40].

### 3.4. Results on the Population Studied

Eight articles showed results on a healthy population [26,29,30,31,32,36,38,39]. Two articles showed results on a chronic NSLBP population [28,33], while five studies showed results on a population with chronic NSLBP and healthy subjects [36,37,38,39,40].

### 3.5. The Quality of the Included Studies

The quality of the included studies was assessed by two investigators (M.C.C and L.L.G). Any disagreement was resolved by consensus or through a third reviewer (S.F.C). One RCT was of high quality [26] and one of moderate quality [27], while the observational studies were of medium-high quality [27,28,30,31,32,33,34,35,36,37,38,39]. The results are shown in Supplementary Sections S4 and S5.

## 4. Discussion

The main objective of this review was to know which muscle synergies of the abdomino-pelvic region (or core) have been studied in healthy subjects and those with chronic NSLBP measured by ultrasound. Knowing how the musculature involving the core behaves together would provide important information on the possible causes or perpetuation of chronic NSLBP with respect to healthy subjects.

Several authors have described the core as a functional unit responsible for the maintenance of lumbo-pelvic stability, in addition to serving as a fundamental axis for the performance of functional tasks involving the upper and lower limbs [41]. This muscle unit would act synergistically, jointly, to provide the stability necessary to perform a task [9]. The musculature of the LAW and of the PF [10] maintains a close relationship with the lumbar, and pubic regions. This musculature, together with the diaphragm [11], has a determining role of mechanical coordination [12] to ensure the stability of the tidal volume and the abdomino-lumbo-pelvic segment. For the action of this musculature of the cavity to be executed in a physiological and adequate manner, it is essential that there is harmony in the totality of the curves of the spine. In addition, the lumbar multifidus muscles are important stabilizers of the spine, and dysfunction of these muscles is associated with low back pain [7,8]. The weakening of the lumbar multifidus muscles leads to impaired spinal function during both dynamic movement and static positioning.

It is hypothesized that any alteration of the functions of any of the elements that compose this functional unit of the core could be the cause of an alteration of the global functioning of this musculature. Thus, this alteration of this global functioning could be the cause or perpetuation of NSLBP.

Despite defending this concept of functional unity, the authors who have tried to explain the functioning of the core have mostly investigated so in a discrete and isolated manner, using techniques such as EMG or MRI (‘Gold standard’) [42]. However, superficial EMG has shown the difficulties in differentiating TrA muscle activity from IO, while deep EMG presents technical difficulties as it is an invasive procedure [41]. Thus, ultrasound has served as an alternative reliable and safe measurement tool to evaluate this musculature [13].

In spite of these limitations, conclusive findings were found that could help researchers and clinicians to better understand the behavior of the musculature in subjects with chronic NSLBP. Miura T et al. [15] found delayed TrA and LM, while other authors [17,18] found LM atrophy in subjects with chronic NSLBP. Excessive activation of the RA, IO, and EO [19] and decreased diaphragm thickness and excursion have also been observed in subjects with chronic NSLBP [20] compared with healthy subjects. In addition, a lack of local activation strategy of the PF musculature has been found in women with chronic NSLBP [21].

Likewise, based on the definition of core as a functional unit, it would be of great interest to know how the four fundamental pillars of core [6] act globally both in healthy subjects and in subjects with chronic NSLBP. In this sense, the use of ultrasound would represent a clear advantage over EMG or MRI as it is a non-invasive procedure that allows the discrimination of each abdominal muscle group effectively with high intraclass correlation indexes with respect to MRI [43].

In relation to the results found in the scientific literature on core muscle synergies, it has been found that most of the studies using ultrasound, analyze the behavior between the muscles of the lateral abdominal wall (TrA, IO and EO), ipsilaterally, or compare it with the contralateral abdominal wall.

Studies that analyzed the behavior between TrA, IO, and OE muscles in subjects with NSLBP agree that they generally deform slower, and fatigue earlier (especially TrA) compared to healthy subjects [36,39]. In another study, they concluded that the IO activates earlier than the TrA or EO in rapid upper limb raising in subjects with NSLBP [34]. However, no studies on this issue have been found in healthy subjects. On the other hand, a ‘normal’ muscle pattern was found by relative resting thickness (cm) RA > IO > EO > TrA and an anticipatory activation strategy of the RA with respect to the TrA in healthy subjects [28,31,33]. However, these results have not been contrasted in subjects with NSLBP.

Few studies evaluate the muscular relationship between the lateral abdominal wall musculature with the rest of the core components. One study has been found that established a synergy between the TrA, IO and EO with the LM, with LM fatigue correlating with the thickness of these three muscles in subjects with NSLBP with respect to healthy subjects [38]. In addition, another study [26] evaluated the LM in relation to the TrA in healthy subjects, showing that invasive intervention on the LM generated changes in TrA thickness. However, this finding has not yet been corroborated in subjects with NSLBP.

In studies that also analyzed the possible asymmetries between the homolateral and contralateral sides of the same muscle in healthy subjects, certain discrepancies have been observed. While Rankin G et al. [31] and Manioon AF et al. [30] found non-statistically significant differences in thickness from 13 to 24%, other authors such as Tahan et al. [33], found statistically significant results in both men and women in TrA, IO, EO (*p* < 0.001) minus RA (*p* = 0.16). Despite the conflicting results, the relationship of this variable to compromised trunk stability has been shown to be null in activities such as the abdominal draw in Maneuver [32,35]. Of note, no studies comparing these results in subjects with chronic NSLBP have been found.

There are limitations in this systematic review as studies using EMG as a measurement tool were not included because it does not allow differentiation between TrA and IO activity. On the other hand, grey literature was not consulted, nor were studies published in a language other than Spanish or English included.

In this systematic review, no studies have been found that analyze the comprehension of all the muscular components in a unique and simultaneous way of the core and relate it or not to NSLBP. Despite this, the synergies that have been found have been described, with the TrA muscle showing the greatest synergies. However, the muscular components of the core, such as the diaphragm or the pelvic floor, have not been studied with respect to other components of this unit, which represents a gap in knowledge. No conclusions can be drawn about how the diaphragm or pelvic floor acts when there is an alteration of the other components of this functional unit. These two muscle groups have been shown to have an important role in trunk stability, as well as in their participation in chronic NSLBP [20,21]. Future lines of research should be aimed at evaluating the symmetry and muscular activity of the core jointly in healthy subjects and in subjects with NSLBP, given its relevance in lumbopelvic stability.

## 5. Conclusions

The synergies studied in this review analyze the behavior between the three muscles that compose the lateral abdominal wall (TrA, IO and EO). In some cases, synergies have been studied this lateral complex and another muscle group of the core, such as the LM and the RA. However, no studies have been found that relate any muscle group to the diaphragm or pelvic floor despite the importance of these two muscle groups in core stability. Most of the studies found are conducted in a healthy population, while only two studies are conducted in a population with chronic NSLBP. More studies are needed to evaluate the behavior of the core as a unit to establish conclusions about its functioning in healthy subjects and those with chronic non-specific low back pain.

## Figures and Tables

**Table 1:** Synergies established among the core muscles measured with ultrasound. (*n* = 9).

Author/Year	Population	Outcome	Core Muscles Synergies
Bialy M,	(*n* = 42)	Onset	TrA, IO, EO
2019 [36]	Healthy and NSLBP		
Dafkou K, 2020 [28]	(*n* = 20)	% change thickness	TrA, RA
	Healthy		
Hoseinpoor TS,	(*n* = 28)	% change thickness	TrA, IO, EO, LM
2015 [38]	Chronic NSLBP and healthy		
Puentedura EJ,	(*n* = 47)	% change thickness	TrA, LM
2017 [26]	Healthy		
Rankin G,	(*n* = 123)	Muscle Pattern	TrA, IO, EO, RA
2006 [31] *	Healthy		
ShahAli S, 2019 [39]	(*n* = 20)	% change thickness	TrA, IO, EO
	Chronic NSLBP and healthy (Women)		
Tahan N, 2016 [33] *	(*n* = 156)	Muscle Pattern	TrA, IO, EO, RA
	Healthy		
Teyhen DS, 2005 [27]	(*n* = 30)	Muscle Pattern	TrA, (IO + EO] **
	Chronic NSLBP		
Westad C, 2010 [34]	(*n* = 24)	Onset	TrA, IO, EO
	Chronic NSLBP		

* Tahan N, 2016 and Rankin G, 2006 showed results for symmetry and synergies between the core muscles. ** (IO + EO): Both muscles represented as an insolate muscle group. + percentage change: % change; transversus abdominis: TrA; internal Oblique: IO; external oblique: EO; rectus abdominis: RA; pelvic floor: PF; diaphragm: DPH; lumbar multifidus: LM; NSLBP: non-specific low back pain.

**Table 2:** Symmetry * measured with ultrasound (Homolateral vs. contralateral) (*n* = 7).

Author/	Population	Outcome	Core Muscles Synergies
Year			
Hides JA, 2009 [37]	(*n* = 39)	% change thickness	TrA, IO
	Chronic NSLBP and healthy		
Mannion AF,	(*n* = 57)	% change thickness	TrA, IO, EO
2008 [30]	Healthy		
Rankin G,	(*n* = 123)	% change thickness	TrA, IO, EO, RA
2006 [31] **	Healthy		
Seo D-K,	(*n* = 41)	% change thickness	TrA, IO, EO
2013 [32]	Healthy		
Tahan N, 2016 [33] **	(*n* = 156)	% change thickness	TrA, IO, EO, RA
	Healthy		
Teyhen DS, 2009 [40]	(*n* = 30)	% change thickness	TrA, IO
	Chronic NSLBP and healthy		
Yoon B, 2018 [35]	(*n* = 18)	% change thickness	TrA, IO, EO
	Healthy (Men)		

* Symmetry is established between the homo and contralateral sides of the same muscle but does not establish a relationship among the muscle groups. ** Tahan N, 2016 and ** Rankin G, 2006 showed results for symmetry and synergies between the core muscles. + percentage change: % change; transversus abdominis: TrA; internal Oblique: IO; external oblique: EO; rectus abdominis: RA; pelvic floor: PF; diaphragm: DPH; lumbar multifidus: LM; NSLBP: non-specific low back pain.

## Supplementary Materials

The following supporting information can be downloaded at: https://www.mdpi.com/article/10.3390/s22228684/s1. Figure S1: Risk of bias Tool (RoB 2.0). Table S1: Search Strategy; Table S2: Studies includied in the systematic review after full reading and data extraction (*n* = 15); Table S3: Causes of exclusion of the excluded studies in the systematic review; Table S4: JBI Critical Appraisal Checklist for case control studies. Obsevational (Case-control studies) (*n* = 5); Table S5: IHE (Institute of Health Economics. Canadian scale). Observational (cases series) Studies (*n* = 7).

## References

[1] Miossenet F., Rose-Dulcina K., Armand S., Genevay S. (2021). A systematic review of movement and muscular activity biomarkers to discriminate non-specific chronic low back pain patients from an asymptomatic population. Sci. Rep..

[2] Oliveira C.B., Maher C.G., Pinto R.Z., Traeger A.C., Lin C.-W.C., Chenot J.-F., van Tulder M., Koes B.W. (2018). Clinical practice guidelines for the management of non-specific low back pain in primary care: An updated overview. Eur. Spine J..

[3] Hartvigsen J., Hancock M.J., Kongsted A., Louw Q., Ferreira M.L., Genevay S., Hoy D., Karppinen J., Pransky G., Sieper J. (2018). What low back pain is and why we need to pay attention. Lancet.

[4] Ehsani F., Arab A.M., Salavati M., Jaberzadeh S., Hajihasani A. (2016). Ultrasound measurement of abdominal muscle thickness with and without transducer fixation during standing postural tasks in participants with and without chronic low back pain: Intrasession and intersession reliability. PM&R.

[5] Ramond A., Bouton C., Richard I., Roquelaure Y., Baufreton C., Legrand E., Huez J.-F. (2011). Psychosocial risk factors for chronic low back pain in primary care—A systematic review. Fam. Pract..

[6] Macedo L.G., Saragiotto B.T., Yamato T.P., Costa L.O., Costa L.C.M., Ostelo R.W., Maher C.G. (2016). Motor control exercise for acute non-specific low back pain. Cochrane Database Syst. Rev..

[7] Huxel-Bliven K.C., Anderson B.E. (2013). Core Stability Training for Injury Prevention. Sports Health.

[8] Sekendiz B., Cuğ M., Korkusuz F. (2010). Effects of Swiss-ball core strength training on strength, endurance, flexibility, and balance in sedentary women. J. Strength Cond. Res..

[9] Sapsford R.R., Hodges P.W., Richardson C.A., Cooper D.H., Markwell S.J., Jull G.A. (2001). Co-activation of the abdominal and pelvic floor muscles during voluntary exercises. Neurourol. Urodyn..

[10] Broyles J.M., Schuenke M.D., Patel S.R., Vail C.M., Broyles H.V., Dellon A.L. (2018). Defining the Anatomy of the Tendinous Intersections of the Rectus Abdominis Muscle and Their Clinical Implications in Functional Muscle Neurotization. Ann. Plast. Surg..

[11] Bordoni B., Marelli F., Bordoni G. (2016). A review of analgesic and emotive breathing: A multidisciplinary approach. J. Multidiscip. Healthc..

[12] Fan C., Fede C., Gaudreault N., Porzionato A., Macchi V., De Caro R., Stecco C. (2018). Anatomical and functional relationships between external abdominal oblique muscle and posterior layer of thoracolumbar fascia. Clin. Anat..

[13] Taghipour M., Mohseni-Bandpei M.A., Behtash H., Abdollahi I., Rajabzadeh F., Pourahmadi M.R., Emami M. (2019). Reliability of Real-time Ultrasound Imaging for the Assessment of Trunk Stabilizer Muscles: A Systematic Review of the Literature. J. Ultrasound Med..

[14] Salvioli S., Pozzi A., Testa M. (2019). Movement Control Impairment and Low Back Pain: State of the Art of Diagnostic Framing. Medicina.

[15] Miura T., Yamanaka M., Ukishiro K., Tohyama H., Saito H., Samukawa M., Kobayashi T., Ino T., Takeda N. (2014). Individuals with chronic low back pain do not modulate the level of transversus abdominis muscle contraction across different postures. Man. Ther..

[16] Keshwani N., McLean L. (2015). Ultrasound Imaging in postpartum women with diastasis recti: Intrarater between-session reliability. J. Orthop. Sports Phys. Ther..

[17] Kiesel K.B., Uhl T.L., Underwood F.B., Rodd D.W., Nitz A.J. (2007). Measurement of lumbar multifidus muscle contraction with rehabilitative ultrasound imaging. Man. Ther..

[18] Hides J., Stanton W., Mendis M.D., Sexton M. (2011). The relationship of transversus abdominis and lumbar multifidus clinical muscle tests in patients with chronic low back pain. Man. Ther..

[19] Blanchard T.W., Smith C., Grenier S.G. (2016). In a dynamic lifting task, the relationship between cross-sectional abdominal muscle thickness and the corresponding muscle activity is affected by the combined use of a weightlifting belt and the Valsalva maneuver. J. Electromyogr. Kinesiol..

[20] Calvo-Lobo C., Almazán-Polo J., Becerro-De-Bengoa-Vallejo R., Losa-Iglesias M.E., Palomo-López P., Rodríguez-Sanz D., López-López D. (2019). Ultrasonography comparison of diaphragm thickness and excursion between athletes with and without lumbopelvic pain. Phys. Ther. Sport.

[21] Thompson J.A., O’Sullivan P.B., Briffa N.K., Neumann P. (2007). Comparison of transperineal and transabdominal ultrasound in the assessment of voluntary pelvic floor muscle contractions and functional maneouvres in continent and incontinent women. Int. Urogynecol. J. Pelvic Floor Dysfunct..

[22] Page M.J., McKenzie J.E., Bossuyt P.M., Boutron I., Hoffmann T.C., Mulrow C.D., Shamseer L., Tetzlaff J.M., Akl E.A., Brennan S.E. (2021). The PRISMA 2020 statement: An updated guideline for reporting systematic reviews. BMJ.

[23] Santos W.M.D., Secoli S.R., Püschel V.A.D.A. (2018). The Joanna Briggs Institute approach for systematic reviews. Rev. Lat. Am. Enferm..

[24] Institute of Health Economics (IHE) (2014). Quality Appraisal of Case Series Studies Checklist.

[25] Sterne J.A.C., Savović J., Page M.J., Elbers R.G., Blencowe N.S., Boutron I., Cates C.J., Cheng H.Y., Corbett M.S., Eldridge S.M. (2019). RoB 2: A revised tool for assessing risk of bias in randomised trials. BMJ.

[26] Puentedura E.J., Buckingham S.J., Morton D., Montoya C., Fernandez-de-las-Penas C. (2017). Immediate changes in resting and contracted thickness of transversus abdominis after dry needling of lumbar multifidus in healthy participants: A randomized controlled crossover trial. J. Manip. Physiol. Ther..

[27] Teyhen D.S., Miltenberger C.E., Deiters H.M., Del Toro Y.M., Pulliam J.N., Childs J.D., Boyles R.E., Flynn T.W. (2005). The use of ultrasound imaging of the abdominal drawing-in maneuver in subjects with low back pain. J. Orthop. Sports Phys. Ther..

[28] Dafkou K., Kellis E., Ellinoudis A., Sahinis C. (2020). The effect of additional external resistance on inter-set changes in abdominal muscle thickness during bridging exercise. J. Sports Sci. Med..

[29] Lee D.Y., Seo D.K. (2012). A comparison of abdominal muscle thicknesses measured by ultrasonography between the abdominal drawing-in and straight leg raise maneuvers. J. Phys. Ther. Sci..

[30] Mannion A.F., Pulkovski N., Toma V., Sprott H. (2008). Abdominal muscle size and symmetry at rest and during abdominal hollowing exercises in healthy control subjects. J. Anat..

[31] Rankin G., Stokes M., Newham D.J. (2006). Abdominal muscle size and symmetry in normal subjects. Muscle Nerve.

[32] Seo D.K., Kim J.S., Lee D.Y., Kwon O.S., Lee S.S., Kim J.H. (2013). The relationship of abdominal muscles balance and body balance. J. Phys. Ther. Sci..

[33] Tahan N., Khademi-Kalantari K., Mohseni-Bandpei M.A., Mikaili S., Baghban A.A., Jaberzadeh S. (2016). Measurement of superficial and deep abdominal muscle thickness: An ultrasonography study. J. Physiol. Anthropol..

[34] Westad C., Mork P.J., Vasseljen O. (2010). Location and sequence of muscle onset in deep abdominal muscles measured by different modes of ultrasound imaging. J. Electromyogr. Kinesiol..

[35] Yoon B., Pyeon H., Kim Y., Hong Y., Lee S. (2018). The relation between abdominal muscle asymmetry and trunk postural stability: An ultrasound imaging study. J. Back Musculoskelet. Rehabil..

[36] Bialy M., Adamczyk W.M., Marczykowski P., Majchrzak R., Gnat R. (2019). Deformations of abdominal muscles under experimentally induced low back pain. Eur. Spine J..

[37] Hides J.A., Belavy D.L., Cassar L., Williams M., Wilson S.J., Richardson C.A. (2009). Altered response of the anterolateral abdominal muscles to simulated weight-bearing in subjects with low back pain. Eur. Spine J..

[38] Hoseinpoor T.S., Kahrizi S., Mobini B., Naji M.A. (2015). Comparison of abdominal muscle thickness changes after a lifting task in subjects with and without chronic low-back pain. Hum. Factors.

[39] ShahAli S., Shanbehzadeh S., ShahAli S., Ebrahimi-Takamjani I. (2019). Application of ultrasonography in the assessment of abdominal and lumbar trunk muscle activity in participants with and without low back pain: A systematic review. J. Manip. Physiol. Ther..

[40] Teyhen D.S., Williamson J.N., Carlson N.H., Suttles S.T., O’Laughlin S.J., Whittaker J., Goffar S.L., Childs J.D. (2009). Ultrasound characteristics of the deep abdominal muscles during the active straight leg raise test. Arch. Phys. Med. Rehabil..

[41] McGill S., Juker D., Kropf P. (1996). Appropriately placed surface EMG electrodes reflect deep muscle activity (psoas, quadratus lumborum, abdominal wall) in the lumbar spine. J. Biomech..

[42] Hodges P.W., Richardson C.A. (1999). Transversus abdominis and the superficial abdominal muscles are controlled independently in a postural task. Neurosci. Lett..

[43] Hides J., Wilson S., Stanton W., McMahon S., Keto H., McMahon K., Bryant M., Richardson C. (2006). An MRI investigation into the function of the transversus abdominis muscle during “drawing-in” of the abdominal wall. Spine.

