# Supplementary material for: Core Synergies Measured with Ultrasound in Subjects with Chronic Non-Specific Low Back Pain and Healthy Subjects: A Systematic Review
Source: Sensors (Basel). 2022 Nov 10;22(22):8684. doi: 10.3390/s22228684 (PMC9692478; doi:10.3390/s22228684)
Supplement: Supplementary file 1 [file sensors-22-08684-s001.zip › sensors-1953483-supplementary.pdf]

Supplementary

Section S1. Search Strategy

Table S1. Search Strategy.

**Project/Study**	**CORE synergies measured with ultrasound in subjects with chronic non-specific low back pain and healthy subjects. A systematic review.**		
**Subproject**	-		
**Requested by**	María Cervera Cano	**Date**	29/Jun/2021

**PICO format with specific terms**	
**DESCRIPTION**	**SCOPE**
**Population**	Healthy subjects and subjects with crhonic nonspecific low back pain
**Intervention**	Ultrasound (M mode, B mode)
**Comparator**	-
**Outcomes**	Muscle synergies (coactivation, activation sequence, activation order, co-contraction, cross-sectional area, muscle thickness)

**Search Filters**	
**Time period**	2005-present
**Aged**	18-60 years old
**TyEO of study**	Randomised Controlled Studies, Observational Studies, Systematic Reviews and Meta-Analyses
**Languages**	English and Spanish
**Others**	Studies measuring muscle synergies with EMG or MRI will be excluded from the search.

**Search terms** (by having been used in similar searches or by literature review)
P – nonspecific low back pain, low back pain, nonspecific low back pain,<br>I – ultrasound, rehabilitative ultrasound, rehabilitative ultrasound imaging (RUSI), ultrasound m mode, ultrasound b mode<br>O – Sinergysm, co contraction, co-activation, thickness, cross sectional area, relation, relationship<br>**abdominal muscles**, external oblique, internal oblique, rectus abdominis pelvic floor, abdominal wall, transversus abdominis, multifidus, lumbar multifidus, diaphragm.
**Databases consulted**
Medline, PEDro, WOS

**Databases used, strategies and results**	
**Database - 1**	
**Database name**	**MEDLINE Ovid**
**Search date**	05/Jul/2021
**Strategy**	Ovid MEDLINE(R) ALL / PubMed(R) <1946 to Present>

	1	Low Back Pain/	23367
	2	low$2 back pain*.kw,tw.	31490
	3	low$2 back ache* .kw,tw.	55
	4	low$2 backache* .kw,tw.	285
	5	LBP.kw,tw.	8865
	6	lumbopelvic pain.kw,tw.	122
	7	(lumbalgia or lumbago).kw,tw.	1596
	8	or/1-7	42336
	9	Ultrasonography/	187054
	10	(ultrasonograph* or ultrasound* or ultrasonic or sonograph* or echograph* or echotomograph*).kw,tw.	432051
	11	or/9-10	491021
	12	exp Abdominal Muscles/	19634
	13	Abdominal Wall/	6847
	14	((abdominal or cremaster or pyramidalis) and muscl*).kw,tw.	18840
	15	((quadratus or transvers* or rectus or recti) and abdomin*).kw,tw.	13756
	16	abdominopelvic muscle*.kw,tw.	2
	17	(obliqu* adj (intern* or extern*)).kw,tw.	439
	18	(pelvic and (diaphragm* or floor)).kw,tw.	11107
	19	'abdominal wall'.kw,tw.	20431
	20	'lumbar multifidus'.kw,tw.	483
	21	or/12-20	66788
	22	Synergis$4.kw,tw.	114665
	23	relation$5.kw,tw.	1907221
	24	Muscle Contraction/	97299
	25	('co-contraction$1' or cocontraction$1 or contraction*).kw,tw.	152542
	26	('co-activit*' or coactivit* or 'co-activation$1' or coactivation$1).kw,tw.	5023
	27	Thickness.kw,tw.	239993
	28	or/22-27	2417169
	29	21 and 28	9796
	30	8 and 11 and 29	250
	31	limit 30 to (yr="2005 -Current" and ("young adult (19 to 24 years)" or "adult (19 to 44 years)" or "middle age (45 to 64 years)") and (english or spanish) and (meta analysis or observational study or randomized controlled trial or "systematic review"))	**41**
**File**	2021.07.05 - Sinergias CORE_MEDLINE_41.docx<br>2021.07.05 - Sinergias CORE_MEDLINE_41.ris		

Database - 2	
**Database name**	**EMBASE**
**Search date**	06/jul/2021
**Strategy**	

Embase Session Results (6 Jul 2021)

No.	Query	Results
#30	#29 AND ('meta analysis'/de OR 'observational study'/de OR 'randomized controlled trial'/de OR 'systematic review'/de) AND ('article'/it OR 'article in press'/it OR 'review'/it)	50
#29	#8 AND #11 AND #27 AND ([english]/lim OR [spanish]/lim) AND [adult]/lim AND [2005-2021]/py	213
#28	#8 AND #11 AND #27	288
#27	#19 AND #26	12584
#26	#20 OR #21 OR #22 OR #23 OR #24 OR #25	3051734
#25	thickness:ti,ab,kw	313884
#24	'co-activit*':ti,ab,kw OR coactivit*:ti,ab,kw OR 'co-activation$':ti,ab,kw OR coactivation$:ti,ab,kw	6054
#23	'co-contraction$':ti,ab,kw OR cocontraction$:ti,ab,kw OR contraction*:ti,ab,kw	195043
#22	'muscle contraction'/de	75656
#21	relation$:ti,ab,kw OR relationship$:ti,ab,kw	2395076
#20	synergis*:ti,ab,kw	174745
#19	#12 OR #13 OR #14 OR #15 OR #16 OR #17 OR #18	79960
#18	'lumbar multifidus':ti,ab,kw	523
#17	'abdominal wall':ti,ab,kw	29121
#16	(pelvic NEAR/5 (diaphragm* OR floor)):ti,ab,kw	19954
#15	(obliqu* NEAR/5 (intern* OR extern*)):ti,ab,kw	3151
#14	(quadratus:ti,ab,kw OR transvers*:ti,ab,kw OR rectus:ti,ab,kw OR recti:ti,ab,kw) AND abdomin*:ti,ab,kw	19920
#13	((abdom* OR cremaster OR pyramidalis) NEAR/5 muscl*):ti,ab,kw	14441
#12	'abdominal wall musculature'/exp	16336
#11	#9 OR #10	1183959
#10	ultrasonograph*:ti,ab,kw OR ultrasound*:ti,ab,kw OR ultrasonic:ti,ab,kw OR sonograph*:ti,ab,kw OR echograph*:ti,ab,kw OR echotomograph*:ti,ab,kw	641065
#9	'echography'/exp	893162
#8	#1 OR #2 OR #3 OR #4 OR #5 OR #6 OR #7	75771
#7	lumbalgia:ti,ab,kw OR lumbago:ti,ab,kw	2252
#6	'lumbopelvic pain':ti,ab,kw	140
#5	lbp:ti,ab,kw	11628
#4	'low* backache*':ti,ab,kw	382
#3	'low* back ache*':ti,ab,kw	138

	#2	'low* back pain*':ti,ab,kw	44677
	#1	'low back pain'/exp	61989
**File**	2021.07.06 - Sinergias CORE_EMBASE_50.docx<br>2021.07.06 - Sinergias CORE_EMBASE_50.riS		

**DATABASE - 3**		
**Database name**	**PEDro**	
**Search date**	06/jul/2021	
**Strategy**	Abstract & Title	"low back pain" ultraso* abdomin* musc*
	Body part	‘Lumbar spine, sacro-iliac jIOnt or pelvis'
	Subdiscipline	‘Musculoskeletal’
	Published since	2005
**File**	2021.07.06 - Sinergias CORE_PEDro_30.doc<br>2021.07.06 - Sinergias CORE_PEDro_30.ris	

Section S2. Studies includied after full reading and data extraction (n=15)

Table S2. Studies includied in the systematic review after full reading and data extraction (n=15).

Author/year	Title	Desing	N	Intervention	Comparator	US	Aims	Synergy	Results
ShahAli S,2019	Ultrasound measurement of abdominal muscles during clinical isometric endurance tests in women with and without low back pain	Observational (Case- control)	(n=20) chronic NSLBP and healthy (women)	(n=10) Isometric trunk lift contraction in supine	(n=10) Bilateral trunk lift manoeuvre of the lower extremities	US. B Mode.	To determine the differences in muscle thickness changes during two manoeuvres. The second objective was to measure the degree of muscle activity of the deep muscles (TRA, IO) compared to the superficial muscles (EO).	TRA, IO,EO	Thickness (cm)
Bialy M, 2019	Deformations of abdominal muscles under experimentally induced LBP	Observational cross-sectional (case-control)	(n=42) Induce LBP and healthy	Mechanical disturbance + induced LBP	Mechanical disturbance	US M mode and TDI	To investigate TDI changes during activation of lateral abdominal muscles under experimentally induced pain.	TRA, IO,EO	Strain rate (ms)
Yoon B,2018	The relation between abdominal muscle asymmetry and trunk postural stability: An ultrasound imaging study.	Observational (Cross-sectional case-series)	(n=18) Healthy (men)	Mechanical stimulation	No comparator	US. B mode.	Compares the muscle thickness between right and left side after a disruptive movement .	TRA, IO,EO,	Thickness (cm), % change thickness

Puentedura EJ, 2017	Immediate Changes in Resting and Contracted Thickness of TRA After DN of LM in Healthy Participants.	RCT	(n=47) Healthy	(n=22) DN in LM	(n=25) DN + Placebo in LM	US. B Mode	To analyse changes in TRA thickness at rest and at maximum concentric contraction after DN of the LM in subjects without LBP.	TRA y LM	CSA of ML and TRA. DN in the LM
Tahan N, 2016	Measurement of superficial and deep abdominal muscle thickness: an ultrasonography study	Observational (cross-sectional case series)	(n=156) Healthy	No intervention	No comparator	RUSI	To determine the thickness and symmetry of the abdominal wall muscles at rest in the Iranian population and to investigate associations with demographic factors (sex, age, BMI...) in order to find reference values.	RA, TRA, IO, EO	Thickness (cm)
Hoseinpoor TS, 2013	A Comparison of Abdominal Muscle Thickness Changes After a Lifting Task in Subjects with and Without Chronic LBP	Observational (cross-sectional case control)	(n=28) Chronic NSLBP and Healthy	No intervention	No comparator	US. B Mode	To compare the effect of standing back extensor muscle fatigue on abdominal muscle recruitment with and without axial loading, in subjects with and without LBP	TRA, IO,EO, LM	% change thickness
Dafkou K, 2013	The Effect of Additional External Resistance on Inter-Set Changes in Abdominal Muscle	Observational (cross-sectional case series)	(n=20) Healthy	8 different exercise conditions during the pelvic bridging position.	No comparator	US. B Mode and TDI.	Identify any differences in the thickness of the abdominal muscles through a series of exercises with an extra load.	RA, TRA	% change thickness

	Thickness during Bridging Exercise								
Seo D-K,2013	The Relationship of Abdominal Muscles Balance and Body Balance	Observational (cross-sectional case series)	(n=41) Healthy	ADIM	No comparator	US. B Mode	To identify the impact on the differences in TRA, IO and EO muscle thickness between left and right sides when performing ADIM.	TRA, IO,EO	Thickness (cm)
Lee D-Y et al, 2012	A Comparison of Abdominal Muscle Thicknesses Measured by US between the ADIM and ASRL Maneuvers	Observational (cross-sectional case series)	(n=37) Healthy	ASRL, ADIM	No comparator	US	To compare and analyse muscle thickness, via RUSI the muscle thickness of the TRA, IO and EO in an ADIM manoeuvre and ASRL, to determine if they are clinically valid functional tests for diagnosis/measurement with sonographic procedures.	TRA, IO y EO	Thickness (cm)
Westad C, 2010	Location and sequence of muscle onset in deep abdominal muscles measured by different modes of ultrasound imaging.	Observational (cross-sectional case series)	(n=24) chronic NSLBP	Elevation of the contralateral arm.	No comparator	US. B Mode	Determine the differences in activation time after a fast arm lift manoeuvre. And determine if it is reliable.	TRA, IO,EO	Time (ms)

Teyhen DS, 2009	Ultrasound Characteristics of the Deep Abdominal Muscles During the ASRL.	Observational (cross-sectional case control)	(n=30)	(n=15) Chronic NSLBP	(n=15) Healthy	US. B Mode	To determine changes in TrA and IO thickness and symmetry with the ASRL manoeuvre in subjects with and without LBP	TRA, IO	% change thickness
Hides JA, 2009	Altered response of the anterolateral abdominal muscles to simulated weight-bearing in subjects with low back pain	Observational (Cross-sectional case-control)	(n=39)	(n=19) chronic NSLBP	(n=20) Healthy	US. B Mode	To compare the automatic recruitment of abdominal muscles between subjects with and without LBP in response to the simulated weight bearing task.	TrA, IO, anterior abdominal fascia of TrA	% change thickness
Mannion AF, 2008	Abdominal muscle size and symmetry at rest and during abdominal hollowing exercises in healthy control subjects.	Observational (cross-sectional case series)	(n=57) Healthy	ADIM	No comparator	US. B mode, M mode	Asymmetry between homo and contralateral sides.<br><br>Comparison by sex and at rest/ADIM maneouvre of TRA, IO and EO muscle thickness.	TRA, IO,EO	Absolute difference between right and left side.
Rankin G,2006	Abdominal muscle size and symmetry in normal subjects.	Observational (cross-sectional case series)	(n=123) Healthy	No intervention	No comparator	RUSI	To provide reference ranges of absolute and relative size and symmetry of healthy abdominal muscles. To examine the relationship between muscle size and anthropology and to	RA, TRA, IO, EO	Thickness (muscle pattern)

							investigate the homogeneity IO,EO and TRA thickness.		
Teyhen DS, 2005	The use of ultrasound imaging of the ADIM in subjetcts with LBP	RCT	(n=28) Chronic NSLBP	(n=13) Traditional exercise + Biofeedback	(n=15) Traditional exercise	RUSI	To characterise the extent to which the ADIM manoeuvre results in preferential activation of the TRA. To determine whether RUSI improves participants' ADIM performance and reliability.	TRA (IO+EO)	Thickness (cm).

Abbreviations: *Nonspecific Low back Pain: NSLBP; Ultrasound: US; Brightness mode: B Mode; Motion Mode: M mode; Transversus abdominis: TRA; Internal Oblique:IO; External Oblique: EO; Centimetres: cm; milliseconds: ms; percentage change in thickness: % change thickness; Low Back Pain: LBP; Tissular Deformation Index: TDI; Randomized Clinical Trials: RCT; Dry Needling: DN; Lumbar Multifidus: LM; Cross Sectional Area: CSA; Rehabilitative Ultrasound Imaging: RUSI; Rectus abdominis: RA; Body Mass Index: BMI; Abdominal Drowing In Maneouver: ADIM; Active Straight Leg Raise: ASRL.*

Section and Topic	Item #	Checklist item	Location where item is reported
**TITLE**			
Title	1	Identify the report as a systematic review.	Page 1
**ABSTRACT**			
Abstract	2	See the PRISMA 2020 for Abstracts checklist.	Page 1
**INTRODUCTION**			

Section and Topic	Item #	Checklist item	Location where item is reported
Rationale	3	Describe the rationale for the review in the context of existing knowledge.	Pages 1-2
Objectives	4	Provide an explicit statement of the objective(s) or question(s) the review addresses.	Page 2
**METHODS**			
Eligibility criteria	5	Specify the inclusion and exclusion criteria for the review and how studies were grouped for the syntheses.	Page 3
Information sources	6	Specify all databases, registers, websites, organisations, reference lists and other sources searched or consulted to identify studies. Specify the date when each source was last searched or consulted.	Page 3
Search strategy	7	Present the full search strategies for all databases, registers and websites, including any filters and limits used.	Page 3<br>Section S1. Table S1. Search Strat-egy
Selection process	8	Specify the methods used to decide whether a study met the inclusion criteria of the review, including how many reviewers screened each record and each report retrieved, whether they worked independently, and if applicable, details of automation tools used in the process.	Page 3
Data collection process	9	Specify the methods used to collect data from reports, including how many reviewers collected data from each report, whether they worked independently, any processes for obtaining or confirming data from study investigators, and if applicable, details of automation tools used in the process.	Page 3
Data items	10a	List and define all outcomes for which data were sought. Specify whether all results that were compatible with each outcome domain in each study were sought (e.g. for all measures, time points, analyses), and if not, the methods used to decide which results to collect.	Pages 3 -4
	10b	List and define all other variables for which data were sought (e.g. participant and intervention characteristics, funding sources). Describe any assumptions made about any missing or unclear information.	Pages 3-4
Study risk of bias assessment	11	Specify the methods used to assess risk of bias in the included studies, including details of the tool(s) used, how many reviewers assessed each study and whether they worked independently, and if applicable, details of automation tools used in the process.	Page 4

Section and Topic	Item #	Checklist item	Location where item is reported
Effect measures	12	Specify for each outcome the effect measure(s) (e.g. risk ratio, mean difference) used in the synthesis or presentation of results.	Pages 3 -4
Synthesis meth-ods	13a	Describe the processes used to decide which studies were eligible for each synthesis (e.g. tabulating the study intervention characteristics and comparing against the planned groups for each synthesis (item #5)).	Page 3
	13b	Describe any methods required to prepare the data for presentation or synthesis, such as handling of missing summary statistics, or data conversions.	Page 3
	13c	Describe any methods used to tabulate or visually display results of individual studies and syntheses.	Pages 5-8
	13d	Describe any methods used to synthesize results and provide a rationale for the choice(s). If meta-analysis was performed, describe the model(s), method(s) to identify the presence and extent of statistical heterogeneity, and software package(s) used.	N/A
	13e	Describe any methods used to explore possible causes of heterogeneity among study results (e.g. subgroup analysis, meta-regression).	N/A
	13f	Describe any sensitivity analyses conducted to assess robustness of the synthesized results.	N/A
Reporting bias assessment	14	Describe any methods used to assess risk of bias due to missing results in a synthesis (arising from reporting biases).	N/A
Certainty assess-ment	15	Describe any methods used to assess certainty (or confidence) in the body of evidence for an outcome.	N/A
**RESULTS**			
Study selection	16a	Describe the results of the search and selection process, from the number of records identified in the search to the number of studies included in the review, ideally using a flow diagram.	Page 4
	16b	Cite studies that might appear to meet the inclusion criteria, but which were excluded, and explain why they were excluded.	Supplementary material. Sec-tion S3. Table S3.
Study	17	Cite each included study and present its characteristics.	Supplementary

Section and Topic	Item #	Checklist item	Location where item is reported
characteristics			material. Section S2. Table S2.
Risk of bias in studies	18	Present assessments of risk of bias for each included study.	Supplementary material. Section S4. Figure S1 Section S5. Table S4 Table S5
Results of individual studies	19	For all outcomes, present, for each study: (a) summary statistics for each group (where appropriate) and (b) an effect estimate and its precision (e.g. confidence/credible interval), ideally using structured tables or plots.	Pages 4-8
Results of syntheses	20a	For each synthesis, briefly summarise the characteristics and risk of bias among contributing studies.	Page 8
	20b	Present results of all statistical syntheses conducted. If meta-analysis was done, present for each the summary estimate and its precision (e.g. confidence/credible interval) and measures of statistical heterogeneity. If comparing groups, describe the direction of the effect.	N/A
	20c	Present results of all investigations of possible causes of heterogeneity among study results.	N/A
	20d	Present results of all sensitivity analyses conducted to assess the robustness of the synthesized results.	N/A
Reporting biases	21	Present assessments of risk of bias due to missing results (arising from reporting biases) for each synthesis assessed.	Page 8
Certainty of evidence	22	Present assessments of certainty (or confidence) in the body of evidence for each outcome assessed.	N/A
**DISCUSSION**			
Discussion	23a	Provide a general interpretation of the results in the context of other evidence.	Page 8-10

Section and Topic	Item #	Checklist item	Location where item is reported
	23b	Discuss any limitations of the evidence included in the review.	Page 10
	23c	Discuss any limitations of the review processes used.	Page 10
	23d	Discuss implications of the results for practice, policy, and future research.	Page 10
**OTHER INFORMATION**			
Registration and protocol	24a	Provide registration information for the review, including register name and registration number, or state that the review was not registered.	Pages 2-3
	24b	Indicate where the review protocol can be accessed, or state that a protocol was not prepared.	Pages 2-3
	24c	Describe and explain any amendments to information provided at registration or in the protocol.	N/A
Support	25	Describe sources of financial or non-financial support for the review, and the role of the funders or sponsors in the review.	Page 10
Competing interests	26	Declare any competing interests of review authors.	Page 10
Availability of data, code and other materials	27	Report which of the following are publicly available and where they can be found: template data collection forms; data extracted from included studies; data used for all analyses; analytic code; any other materials used in the review.	Supplementary material

From: Page MJ, McKenzie JE, Bossuyt PM, Boutron I, Hoffmann TC, Mulrow CD, et al. The PRISMA 2020 statement: an updated guideline for reporting systematic reviews. BMJ 2021;372:n71. doi: 10.1136/bmj.n71

For more information, visit: http://www.prisma-statement.org/

Section S3. Studies excluded after full reading and causes (n=50)

Table S3. Causes of exclusion of the excluded studies in the systematic review

**1. Cause of exclusion: Measure several muscles before and after in isolation (n=38)**	
**Author/year**	**Article**
Akbari A, 2008	The effect of motor control exercise versus general exercise on lumbar local stabilizing muscles thickness: randomized controlled trial of patients with chronic low back pain
Amerijckx C, 2020	Influence of phase of respiratory cycle on ultrasound imaging of deep abdominal muscle thickness
Batıbay S, 2020	Effect of Pilates mat exercise and home exercise programs on pain, functional level, and core muscle thickness in women with chronic low back pain
Beazell JR, 2011	Changes in lateral abdominal muscle thickness during an abdominal drawing in Maneouver in individuals with and without low back pain
Cho M, 2015	The effects of bridge exercise with the abdominal drawing-in maneuver on an unstable surface on the abdominal muscle thickness of healthy adults
Debuse D, 2013	Low impact weight-bearing exercise in an upright posture increases the activation of the two key local muscles on the lumbopelvic region.
Djordjevic O, 2015	Relationship Between Electromyographic Signal Amplitude and Thickness Change of the Trunk Muscles in Patients with and Without Low Back Pain
Gala-Alarcon P, 2018	Ultrasound Evaluation of the Abdominal Wall and Lumbar Multifidus Muscles in Participants Who Practice Pilates: A 1-year Follow-up Case Series
Gibons, 2019	Exercising on Different Unstable Surfaces Increases Core Abdominal Muscle Thickness: An Observational Study Using Real-Time Ultrasound
Gong W, 2016	The effects of running in place in a limited area with abdominal drawing-in maneuvers on abdominal muscle thickness in chronic low back pain patients
Gong W,2015	Effects of bridge exercises with a sling and vibrations on abdominal muscle thickness in healthy adults
Guthrie, 2012	The effect of traditional bridging or suspension-exercise bridging on lateral abdominal thickness in individuals with low back pain
Halliday MH, 2019	A randomized clinical trial comparing the McKenzie method and motor control exercises in people with chronic low backpain and a directional preference: 1 year follow-up
Huang Q, 2014	Comparison of the Efficacy of Different Long-term Interventions on Chronic Low Back Pain Using the Cross-sectional Area of the Multifidus Muscle and the Thickness of the Transversus Abdominis Muscle as Evaluation Indicators
Kiesel KB, 2007	A Comparison of Select Trunk Muscle Thickness Change Between Subjects With Low Back Pain Classified in the Treatment-Based Classification System and Asymptomatic Controls
Kiesel KB, 2008	Rehabilitative ultrasound measurement of select trunk muscle activation during induced pain
Kim H, 2008	Effect of whole body horizontal vibration exercise in chronic low back pain patients: Vertical versus horizontal vibration exercise
Kim HI,2012	Comparison of Changes in Abdominal Muscle Thickness Using Ultrasound Imaging during the Abdominal Drawing-in Maneuver Performed by Patients with Low Back Pain and Healthy Subjects

Kim J-S,2018	Abdominal draw-in maneuver combined with simulated weight bearing increases transversus abdominis and internal oblique thickness
Kong Y-S,2015	The effects of prone bridge exercise on trunk muscle thickness in chronic low back pain patients
Koppenhaver SL ,2011	Association Between Changes in Abdominal and Lumbar Multifidus Muscle Thickness and Clinical Improvement After Spinal Manipulation
Lariviere C,2019	Ultrasound Measures of the Abdominal Wall in Patients with Low Back Pain Before and After an 8-week Lumbar Stabilization Exercise Program, and Their Association With Clinical Outcomes
Lee J-Y,2018	The effect of therapeutic abdominal drawing-in maneuver using ultrasonography on lateral abdominal muscle thickness and balance
Lee K,2017	Comparison of lateral abdominal muscle thickness during bridge exercises with different support surfaces in healthy individuals
Lee SH,2014	The effect of abdominal bracing in combination with low extremity movements on changes in thickness of abdominal muscles and lumbar strength for low back pain
Madokoro S, 2020l	Effect of the Abdominal Draw-In Maneuver and Bracing on Abdominal Muscle Thickness and the Associated Subjective Difficulty in Healthy Individuals
Mew R,2009	Comparison of changes in abdominal muscle thickness between standing and crook lying during active abdominal hollowing using ultrasound imaging
Nabavi N, 2018	The Effect of 2 Different Exercise Programs on Pain Intensity and Muscle Dimensions in Patients with Chronic Low Back Pain: A Randomized Controlled Trial
Nagai H, 2016	Deep abdominal muscle thickness measured under sitting conditions during different stability tasks
Pinto RZ, 2011	The effect of lumbar posture on abdominal muscle thickness during an isometric leg task in people with and without non-specific low back pain
Rasouli O,2020	The Effect of Respiratory Phase on Abdominal Muscle Activity During Stable and Unstable Sitting Positions in Individuals with and Without Chronic Low Back Pain
Sugaya T, 2014	Ultrasound Evaluation of Muscle Thickness Changes in the External Oblique, Internal Oblique, and Transversus Abdominis Muscles Considering the Influence of Posture and Muscle Contraction
Sutherlin MA, 2018	Changes in Muscle Thickness Across Positions on Ultrasound Imaging in Participants with or Without a History of Low Back Pain
Teyhen Ds, 2008	Changes in Deep Abdominal Muscle Thickness During Common Trunk-Strengthening Exercises Using Ultrasound Imaging
Vasseljen O, 2010	Abdominal muscle contraction thickness and function after specific and general exercises: A randomized controlled trial in chronic low back pain patients
Winnard A, 2017	Movement amplitude on the Functional Re-adaptive Exercise Device: deep spinal muscle activity and movement control
Yang H-S, 2013	Changes in the Thickness of Trunk Stabilizer Muscles According to Increased Lifting Loads in Stoop Lifting
Yang H-S,2018	Difference of the thickness and activation of trunk muscles during static stoop lift at different loads between subjects with and without low back pain

2. Cause of exclusion: Other population (n=3)

**Author/year**	**Article**

Cai C, 2015	Low Back and Lower-Limb Muscle Performance in Male and Female Recreational Runners with Chronic Low Back Pain
Ehsani, 2020	Stabilization exercise affects function of transverse abdominis and pelvic floor muscles in women with postpartum lumbo-pelvic pain: a double-blinded randomized clinical trial study
Stuge B, 2006	Abdominal and pelvic floor muscle function in women with and without long lasting pelvic girdle pain
**3. Cause of exclusion: Another objective (n=2)**	
**Author/year**	**Article**
Tahan N, 2013	Relationship between ultrasonography and electromyography measurement of abdominal muscles when activated with and without pelvic floor muscles contraction
Tahan N, 2014	Reliability of the ultrasound measurements of abdominal muscles activity when activated with and without pelvic floor muscles contraction
**4. Cause of exclusion: Other type of relationship established with US (n=5)**	
**Author/year**	**Article**
Hebert JJ, 2010	The Relationship of Transversus Abdominis and Lumbar Multifidus Activation and Prognostic Factors for Clinical Success with a Stabilization Exercise Program: A Cross-Sectional Study
Ota M,2011	Differences in Abdominal Muscle Thicknesses between Chronic Low Back Pain Patients and Healthy Subjects
Sutherlin MA,2019	Correlations Between Anthropometric Measures and Muscle Thickness Using Ultrasound Imaging
Teyhen DS ,2012	Abdominal and Lumbar Multifidus Muscle Size and Symmetry at Rest and During Contracted States Normative Reference Ranges
Whittaker JL,2013	Comparison of the Sonographic Features of the Abdominal Wall Muscles and Connective Tissues in Individuals with and Without Lumbopelvic Pain
**5. Cause of exclusion: Results for one muscle only (n=2)**	
**Author/year**	**Article**
Unsgaard-Tondel M, 2012	Is activation of transversus abdominis and obliquus internus abdominis associated with long-term changes in chronic low back pain? A prospective study with 1-year follow-up
Vasseljen O, 2012	Effect of core stability exercises on feed-forward activation of deep abdominal muscles in chronic low back pain: A randomized controlled trial

Section S4. Quality of the studies included for RCT

Figure S1. Risk of bias Tool (RoB 2.0)

RCTs (n=2).

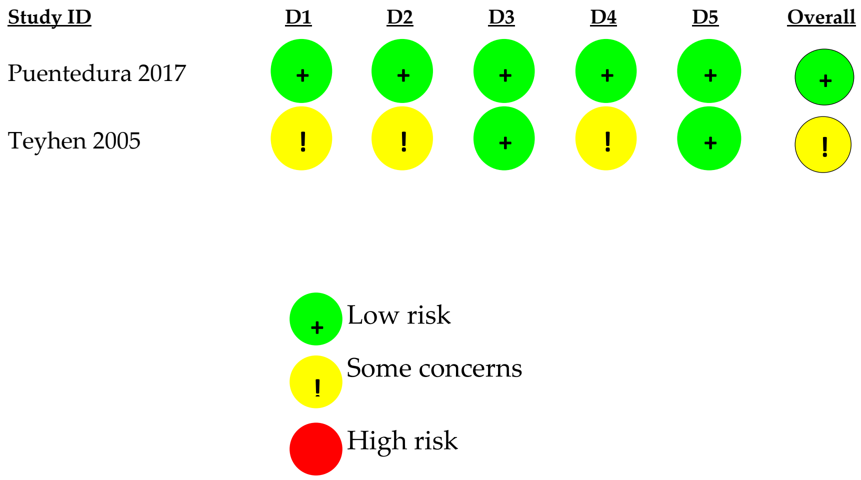

Section S5. Quality of the studies included for Observational studies

Table S4. JBI Critical Appraisal Checklist for case control studies. Obsevational (Case-control studies) (n=5).

JBI CRITICAL APPRAISAL CHECKLIST FOR CASE CONTROL STUDIES	Hides 2009	Hosseinpoor 2015	ShahAli 2018	Teyhen 2009	Bialy 2019
1. Were the groups comparable other than the presence of disease in cases or the absence of disease in controls?	Y	Y	Unclear	Y	Y
2. Were cases and controls matched appropriately?	Y	Y	Y	Y	Y
3. Were the same criteria used for identification of cases and controls?	Y	Y	N	Y	Y
4. Was exposure measured in a standard, valid and reliable way?	Y	Y	Y	Unclear	Y
5. Was exposure measured in the same way for cases and controls?	Y	Y	Y	Y	Y
6. Were confounding factors identified?	N	N	Unclear	N	N
7. Were strategies to deal with confounding factors stated?	N	Unclear	Unclear	Unclear	Unclear

8. Were outcomes assessed in a standard, valid and reliable way for cases and controls?	Y	Y	Y	Y	Y
9. Was the exposure period of interest long enough to be meaningful?	Y	Y	Y	Y	Y
10. Was appropriate statistical analysis used?	Y	Y	Y	Y	Y

Table S5. IHE (Institute of Health Economics. Canadian scale). Observational (cases series) Studies (n=7).

**Study objective**	**Mannion 2008**	**Dafkou 2020**	**Rankin 2006**	**Seo 2013**	**Yoon 2018**	**Tahan 2016**	**Westad 2010**
1. Was the hypothesis/ aim/objective of the study clearly stated?	P	Y	Y	Y	Y	P	P
**Study design**							
2. Was the study conducted prospectively?	Y	U	Y	Y	Y	Y	Y
3. Were the cases collected in more than one centre?	Y	N	Y	U	U	U	U
4. Were patients recruited consecutively?	Y	U	Y	Y	Y	Y	Y
**Study population**							
5. Were the characteristics of the patients included in the study described?	Y	Y	Y	P	Y	Y	P
6. Were the eligibility criteria (i.e. inclusion and exclusion criteria) for entry into the study clearly stated?	P	Y	Y	Y	Y	Y	Y
7. Did patients enter the study at a similar point in the disease?	Y	Y	Y	Y	Y	Y	Y
**Intervention and co-intervention**							
8. Was the intervention of interest clearly described?	Y	Y	Y	Y	Y	Y	Y
9. Were additional interventions (co-interventions) clearly described	Y	Y	Y	Y	Y	Y	Y
**Outcome measure**							

10. Were relevant outcome measures established a priori?	Y	Y	Y	Y	Y	Y	Y
11. Were outcome assessors blinded to the intervention that patients received?	N	N	N	U	N	U	U
12. Were the relevant outcomes measured using appropriate objective/subjective methods?	Y	Y	Y	Y	Y	Y	Y
13. Were the relevant outcome measures made before and after the intervention?	Y	Y	Y	Y	Y	Y	Y
**Statistical analysis**							
14. Were the statistical tests used to assess the relevant outcomes appropriate?	Y	Y	Y	Y	Y	Y	Y
**Results and conclusions**							
15. Was follow-up long enough for important events and outcomes to occur?	U	U	Y	U	U	U	U
16. Were losses to follow-up reported?	N	N	N	N	U	U	N
17. Did the study provided estimates of random variability in the data analysis of relevant outcomes?	Y	Y	Y	Y	Y	Y	Y
18. Were the adverse events reported?	N	N	N	N	N	N	Y
19. Were the conclusions of the study supported by results?	Y	Y	Y	Y	Y	Y	N
**Competing interests and sources of support**							
20. Were both competing interests and sources of support for the study reported?	Y	Y	Y	Y	Y	Y	Y

- ***Y:** Yes*
- ***N:** No*
- ***P:** Parcial*
- ***U:** Unclear*
